# Cognitive impairment in multiple system atrophy: Changing concepts

**DOI:** 10.1590/S1980-57642011DN05040008

**Published:** 2011

**Authors:** Agessandro Abrahão, Livia Almeida Dutra, Pedro Braga Neto, José Luiz Pedroso, Ricardo Araújo de Oliveira, Orlando Graziani Povoas Barsottini

**Affiliations:** Division of General Neurology and Ataxias, Department of Neurology and Neurosurgery, Universidade Federal de São Paulo, São Paulo SP, Brazil.

**Keywords:** multiple system atrophy, cognitive impairment, dementia, atypical parkinsonism, neuropsychological evaluation

## Abstract

Multiple system atrophy (MSA) is characterized by a variable combination of cerebellar ataxia, parkinsonism and pyramidal signs associated with autonomic failure. Classically, cognitive impairment was not considered a clinical feature of MSA and dementia was pointed out as an exclusion diagnostic criteria. Based on comprehensive neuropsychological assessment, cognitive impairment was found to be a frequent feature in MSA, and clinically-defined dementia is now reported in 14-16% of cases. This article reviews the current data on cognitive impairment in MSA along with its neuropsychological profile and pathophysiology.

## Abstract (pt)

A Atrofia de Múltiplos Sistemas (AMS) é caracterizada por uma combinação variável de ataxia cerebelar, parkinsonismo e sinais piramidais, associados à disfunção autonômica. Classicamente, o comprometimento cognitivo não é considerado como manifestação clínica da AMS e a demência é apontada como um critério de exclusão para o diagnóstico. Baseado na avaliação neuropsicológica ampla, o comprometimento neuropsicológico revelou-se frequente na AMS e a presença de demência é atualmente descrita em 14-16% dos casos. Este artigo revisa os dados atuais sobre o comprometimento cognitivo na AMS, seu perfil neuropsicológico e fisiopatologia.

## Introduction

Multiple system atrophy (MSA) is characterized by a variable combination of cerebellar ataxia, parkinsonism and pyramidal signs associated with autonomic failure. The core feature of MSA pathology is the presence of widespread argyrophilic neuronal and glial cytoplasmatic inclusions (GCI) containing α-synuclein, which is the obligatory feature for a definite diagnosis. The density of GCIs containing α-synuclein correlates significantly with neuronal deterioration and disease duration. Also, another common pathological finding is significant neuronal loss in the basal ganglia, cerebellum, pons, inferior olivary nuclei and spinal cord.^1-3^

The earliest description of sporadic cases presenting with cerebellar ataxia and urinary incontinence dates from 1900 and was reported by Dejerine and Thomas. They introduced the term olivopontocerebellar atrophy for these neurological conditions.^4^ In the years that followed, the concepts of postural hypotension and dysautonomia were incorporated by Shy and Drager, who described a syndrome characterized by orthostatic hypotension, anhidrosis, urinary and fecal incontinence, as well as sexual impotence associated with variable neurological involvement.^5,6^ In the 1960s, cases presenting predominantly with parkinsonism were described as striatonigral degeneration.^7,8^ Finally, the term MSA was proposed by Graham and Oppenheimer to encompass the three conditions above.^9^

The first diagnostic criteria^10^ have indicated that dementia was an exclusion criteria for the diagnosis of MSA. The ensuing updated consensus of 2008^11^ considered dementia (as defined by DSM-IV^12^) and severe cognitive impairment (according to the Mini Mental State Examination - MMSE^13^) as non-supporting features. However, in the last few years, cognitive impairment was found to be a frequent feature in MSA based on evidence from qualitative neuropsychological assessment.^14,15^ Dementia in MSA is now reported in 14-16% of cases.^14,16^

In the present report, we review and discuss the current data on cognitive impairment in MSA.

## Methods

A literature search was carried out on the PubMed database analyzing only papers published in English and indexed on Medline up until May, 2011. The following key words were used: multiple system atrophy, striatonigral degeneration, olivopontocerebellar atrophy, memory, cognitive impairment and dementia. From the retrieved articles, a total of 40 papers were included for relevance and encompassed retrospective and prospective studies, case series reports, clinical trials and reviews.

### MSA overview

MSA comprises two categories of clinical manifestations: MSA with predominant parkinsonism (MSA-P) and MSA with predominant cerebellar ataxia (MSA-C).^11^ According to the final report of the European Multiple System Atrophy Study Group (EMSA-SG),^17^ mean age at disease onset was 57.8 years and disease duration at time of diagnosis was 5.8 years. MSA-P represented 68.2% of European patients and is also more common in the North America.^18^ By contrast, in Japan, MSA-C is more prevalent than MSA-P (83.8% *vs.* 16.2% in a cohort of 142 patients^19^ and 67.4% *vs.* 32.6% in a retrospective analysis of 230 Japanese patients).^20^

A consensus statement on the diagnosis of MSA was proposed in 1998^10^ and revised in 2008 by Gilman et al.^11^ The latter simplified the previous criteria and incorporated new pathological, laboratory and neuroimaging findings.

Definite MSA diagnosis requires the demonstration of widespread α-synuclein-positive GCI and neurodegenerative findings in striatonigral or olivopontocerebellar structures. Probable and possible MSA criteria are listed in Table 1.^11^ Additionally, supporting and non-supporting features for MSA diagnosis are also summarized in Table 1. Of note, dementia is considered a non-supporting feature.^11^

**Table 1:** Criteria for probable and possible MSA and supporting and non-supporting features. Adapted from Gilman, 2008.

Criteria for probable MSA			Criteria for possible MSA			Supporting features	Non-supporting features
Autonomic failure involving urinary incontinence (inability to control the release of urine from the bladder, with erectile dysfunction in males) or an orthostatic decrease of blood pressure within 3 min of standing by at least 30 mm Hg systolic or 15 mm Hg diastolic			Poorly levodopa-responsive parkinsonism (bradykinesia with rigidity, tremor, or postural instability)	OR	A cerebellar syndrome (gait ataxia with cerebellar dysarthria, limb ataxia, or ce­rebellar oculomotor dysfunction)	• Orofacial dystonia • Disproportionate anterocollis • Camptocormia (severe anterior flexion of the spine) and/or Pisa syndrome (severe lateral flexion of the spine) • Contractures of hands or feet • Inspiratory sighs • Severe dysphonia • Severe dysarthria • New or increased snoring • Cold hands and feet • Pathologic laughter or crying • Jerky, myoclonic postural/action tremor	• Classic pill-rolling rest tremor • Clinically significant neuropathy • Hallucinations not induced by drugs • Onset after age 75 y • Family history of ataxia or parkinsonism • Dementia (by on DSM-IV criteria) • White matter lesions suggesting multiple sclerosis
AND			AND				
Poorly levodopa-responsive parkinsonism (bradykinesia with rigidity, tremor, or postural instability)	OR	A cerebellar syndrome (gait ataxia with cerebellar dysarthria, limb ataxia, or ce­rebellar oculomotor dysfunction)	At least one feature suggesting autonomic dysfunction (otherwise unexplained urinary urgency, frequency or incomplete bladder emptying, erectile dysfunction in males, or significant orthostatic blood pressure decline that does not meet the level required in probable MSA)				
			AND				
			At least one of the additional features: • Possible MSA-P or MSA-C - Babinski sign with hyperreflexia - Stridor • Possible MSA-P - Rapidly progressive parkinsonism - Poor response to levodopa - Postural instability within 3 y of motor onset - Gait ataxia, cerebellar dysarthria, limb ataxia, or cerebellar oculomotor dysfunction - Dysphagia within 5 y of motor onset - Atrophy on MRI of putamen, middle cerebellar peduncle, pons, or cerebellum - Hypometabolism on FDG-PET in putamen, brainstem, or cerebellum • Possible MSA-C - Parkinsonism (bradykinesia and rigidity) - Atrophy on MRI of putamen, middle cerebellar peduncle, or pons - Hypometabolism on FDG-PET in putamen - Presynaptic nigrostriatal dopaminergic denervation on SPECT or PET				

Parkinsonism in MSA shares similar clinical features to Parkinson's disease (PD), such as bradykinesia, rigidity, postural instability and also resting tremor. Transitory response to levodopa may also be seen. On the other hand, when cerebellar syndrome is present, ataxic gait with early falls and ocular movement abnormalities are found.^21^ Pyramidal signs occur in almost fifty percent of patients^22^ and dysphagia and dysarthria may appear in early stages.^23^ Dysautonomia may cause erectile dysfunction in male patients, urinary incontinence, constipation, orthostatic blood pressure decline, diaphoresis or cold blue hands.^21^ Dystonia is a supporting feature, especially when anterocollis or Pisa syndrome (axial dystonia with lateral flexion of trunk) are present.^3, 21^

In MSA-P, MRI findings include putaminal atrophy, putaminal dorsolateral T2-hypointensity, with "slit-like" marginal T2-hyperintensity. In MSA-C, MRI findings include atrophy of the lower brainstem, pons, middle cerebellar peduncles, and vermis, as well as pontine cruciform hyperintensity on T2-weighted images and diffuse T2-hyperintensity of middle cerebellar peduncles.^24^

### Cognitive impairment in MSA

**Early reports** - Classically, dementia was considered an exclusion criteria based on analysis of retrospective data.^10,15,18,25^ In this context, MSA diagnosis was ruled out at the first medical interview if significant cognitive impairment was present although no reliable cognition analyses is available from these studies.^15^

Nevertheless, in the early nineties, a few authors reported the occurrence of cognitive impairment in patients with sporadic late-onset ataxia or parkinsonism associated with dysautonomia. In this scenario, Sullivan et al.^26^ reported a 55-year-old woman that presented clinical and neuroimaging features characteristic of MSA-P concomitant with short term memory and language impairment. Consistent data on this issue emerged one year later, when neuropsychological evaluation of sixteen MSA patients disclosed frontal lobe dysfunctions compared to matched controls.^27^

**Epidemiology** - The frequency of cognitive impairment in MSA patients is highly variable, as are the methods and tools employed for its diagnosis, ranging from bedside clinical impression to comprehensive neuropsychological battery. In a retrospective analysis of 203 pathologically-confirmed MSA patients, dementia was found in only one patient, although mild or moderate intellectual impairment was documented in 25% of patients, based on bedside unstructured impressions.^22^

Results from a Japanese center reported that dementia was found in 10 out of 58 MSA patients evaluated. Although dementia may occur as a late feature of the disease, one Japanese patient presented dementia one-year prior to motor symptoms. Unfortunately no post-mortem examination was performed to confirm a definite diagnosis.^27^ Nevertheless, disease duration correlated weakly with cognitive impairment.

In another retrospective study of pathologically-confirmed cases, dementia was identified in 15.7% of 38 MSA patients, typically five years after disease onset.^14^ O'Sullivan et al.^16^ demonstrated that 11 patients out of 83 MSA cases were demented, using DSM-IV criteria. The final follow-up analysis of the prospective European cohort (EMSA-SG) published in 2010 revealed that 4.5% of the patients presented dementia based on MMSE evaluation.^17^ Unfortunately, the MMSE probably underestimated the prevalence of cognitive impairment in this cohort. More substantial evidence that cognitive impairment is consistent with a diagnosis of MSA was found in a prospective neuropsychological analysis of 372 MSA patients published by Brown et al.^15^, in a subgroup analysis of the NNIPPS study (Neuroprotection and Natural History in Parkinson-Plus Syndromes - a randomized, multi-center, double-blind, placebo-controlled study of the efficacy and safety of riluzole in patients with MSA and Progressive Supranuclear Palsy - PSP). In this latter study, the Mattis Dementia Rating Scale (DRS), a broader and more comprehensive neuropsychological battery, disclosed that 19.6% of MSA patients presented significant cognitive impairment. A single cognitive domain impairment was observed in 28.6%, and multiple domain impairment in 13.5%, of cases. Moreover, using the Frontal Assessment Battery (FAB), 31.8% of the patients scored below the cut-off score. Logistic regression revealed that severe motor disability, fewer than 10 years of education, male gender, cardiovascular dysautonomia and absence of genitourinary symptoms were considered predictors of cognitive impairment. This is the largest prospective cohort of patients with MSA studied to date and indicates that a remarkable proportion of these patients have significant cognitive impairment.

**Neuropsycological profile in MSA and other parkinsonian syndromes** - MSA, PD, and Dementia with Lewy Bodies (DLB) are called synucleinopathies because they share α-synuclein inclusions as a neuropathological hallmark. In this group, common clinical features besides parkinsonism, include REM (rapid eye movement) sleep behavior disorder,^28^ dysautonomia symptoms and cognitive impairment, which are well known in early DLB and late PD. When comparing cognitive profiles of synucleinopathies, it is interesting to note that cognitive impairment is more pronounced in DLB and only mild in Parkinson's disease, whereas MSA patients present an intermediate profile.^29^

Impaired cognitive domains in PD include attention, memory, visuo-spatial, constructional, and executive functions. Only PD and DLB patients showed a pattern of severe global impairment in memory and executive function.^30^ On the other hand, MSA patients present involvement of language, non-verbal reasoning, working memory and attention, according to previous studies. There are conflicting data regarding the involvement of visuospatial function and praxis in MSA.^29,31-38^

Patients with MSA-P present severe involvement of visuospatial and constructional function, verbal fluency, and executive function, while MSA-C patients present milder degree of impairment only in visuospatial and constructional function.^41^ In addition, neuropsychological impairment in patients with MSA-P was significantly correlated with a decrease in prefrontal perfusion. However, Burk et al.^39^ demonstrated impaired verbal memory and verbal fluency in 20 MSA-C patients. Despite conflicting data, studies suggest differences in cognitive impairment between MSA-P and MSA-C patients while those patients with MSA-P tend to exhibit broader and more severe impairment in cognitive function compared with patients that have MSA-C.^40^

Neuropsychological performance in MSA-C, one of the causes of olivopontocerebellar atrophy (OPCA), was compared to other causes of OPCA. In this study, four groups were compared: controls, MSA-C, ataxic patients without autonomic failure and negative family history (called sporadic OPCA in the cited study) and positive family history of dominantly inherited OPCA. The MSA-C group scored the lowest results, suggesting that an intrinsic physiopathological mechanism is present in MSA-C, compared to other causes of OPCA.^31^

Progressive Supranuclear Palsy (PSP) constitutes another differential diagnosis of MSA and its typical presentation is characterized by parkinsonism, dystonia, bulbar signs and ocular abnormalities such as ophthalmoparesis. Cognitive function in PSP may be affected in up to 60% of cases, with prominent deficits in attention and executive function, with verbal fluency being severely affected, as well as deficits in both verbal and non-verbal memory with a relative preservation of recognition.^15^ When comparing MSA and PSP using different instruments such as the FAB, MMSE and Mattis Dementia rating Scale, the profile observed in MSA and PSP were similar, albeit more severe in patients with PSP.^15^

More recently, the general concept of cognitive impairment in parkinsonian syndromes is that, independent of the underlying pathology, a core pattern of cognitive impairment occurs, probably reflecting both cortical and subcortical atrophy and their associated cortical pathological changes.^15^ Neuropsychological assessment serves an important role in providing objective evidence of cognitive impairment to support the clinical diagnosis of dementia. However, its role in differential diagnosis is not conclusive.^30^

**Pathophysiology** - The occurrence of cognitive impairment and dementia in synucleinopathies is well known. Although DLB, PD and MSA share α-synuclein inclusions in their pathophysiology, clinical and neuropsychologic differences are explained by the deposition of these inclusions in variable regions and structures of the brain in each disorder. In PD, Lewy bodies and neurites are found early in the deep brainstem and substantia nigra. Conversely, paralimbic and neocortical structures are affected early in DLB. Finally, in MSA the deep brainstem, cerebellar nuclei, striatal and basal ganglia regions show α-synuclein-staining inclusions.^32^ Moreover, α-synuclein inclusions are found in neurons in PD and DLB, while in MSA both neuronal intranuclear inclusions and glial cytoplasmic inclusions are present.^1,3,32^

The α-synuclein deposition in the striatonigral pathway might justify the occurrence of cognitive impairment in MSA-P, since prefrontal cortex receives afferences from ganglia basal.^33^ On the other hand, the recently described role of cerebellum in cognitive tasks such as working memory,^34^ executive function,^35^ verbal memory,^36^ language,^37^ and attention^38^ offers some explanation for cognitive impairment in patients with MSA-C. Indeed, disruption of cerebellar circuits to prefrontal, posterior parietal, superior temporal and limbic cortices leads to cognitive and behavioral deficits described as Cerebellar Cognitive Affective Syndrome (CCAS), characterized by executive, visuospatial, linguistic and affective disturbances.^38^

Kawamura et al. hypothesized that cognitive impairment in MSA could be influenced by either cerebral hypoperfusion associated with orthostatic hypotension or nocturnal hypoxemia caused by sleep-disordered breathing (SDB), both of which are important clinical symptoms observed in MSA patients. In the cited study however, this hypothesis could not be proven.^29^

**Neuropsycological scales** - Neuropsychological scales used in the evaluation of cognitive impairment in MSA are highly heterogeneous among published studies. A list of neuropsychological tests used in MSA cognitive evaluation is summarized on Table 2.

**Table 2:** Neuropsychological impairment in MSA.

Study	Significantly Impaired Performance	Neuropsychological Tests Performed
Pillon et al., 1995	Executive function	MMSE; Mattis Dementia Rating Scale (DRS); Wechsler adult intelligence scale (WAIS); Raven progressive matrices; Wechsler memory scale (WMS); simplified version of the Wisconsin card sorting test (WCST); semantic fluency test (names of animals in one minute), phonemic fluency test (words beginning with M in one minute); California verbal learning test; Grober and Buschke’s test.
Meco et al., 1996	Attention Tasks	Trail making test; Stroop word color test; verbal fluency test; Rey auditory verbal learning test (RAVLT); Conceptual thinking WCST; Raven progressive matrices.
Soliveri et al., 2000	Verbal fluency test	MMSE; Raven progressive matrices; short tale test for long- term verbal memory; phonemic verbal fluency test; visual search test; visuospatial orientation line test of Benton; Nelson modification of the WCST.
Dujardin et al., 2003	Executive Function	Phonemic and semantic fluency task; Spatial sequences generation task; Nelson modification of the WCST; Stroop word color test.
Lange et al., 2003	Verbal fluency, problem solving and verbal and figural working memory	Simple phonemic verbal fluency task (words beginning with S in one minute); simple semantic verbal fluency task (names of animals in one minute); alternate phonemic fluency task (H/T-Word-Test); alternating phonemic fluency task (Sport/Fruit-Test); Digit Span Forward task; Digit Span Backward task; Visual Working Memory Test; Verbal Recency Task; Tower of London task.
Paviour et al., 2005	Executive Function	MMSE; DRS; Frontal assessment battery (FAB).
Bak et al., 2005	Global cognition	MMSE, DRS, Addenbrooke’s cognitive examination (ACE).
Bürk et al., 2006	MSA-C: verbal memory and verbal fluency	MMSE, Similarities and Picture Completion subtests of a short version of the German version of the Wechsler Adult Intelligence Scale (WIP), Digit Span Forward task; Digit Span Backward task, WMS, Rey-Osterrieth Complex Figure, WCST, semantic fluency test (names of countries in one minute), phonemic fluency test(words beginning with B in one minute).
Kawai et al., 2008	MSA-P: visuospatial and constructional function, verbal fluency, and executive function. MSA-C: visuospatial and constructional function (milder)	MMSE, Digit Span, Revised Wechsler Memory Scale, semantic fluency (animals in one minute), phonemic fluency (Japanese nouns starting with the Japanese Kana character "Ka" in one minute), WCST, Rule Shift Cards test of the Behavioral Assessment of the Dysexecutive Syndrome, revised WAIS.
Sachin et al., 2008		
Kao et al., 2009	Executive Function, visuospatial skills	MMSE, modified Trails B, Stroop word color test, trial 1 of the Design Fluency subtest of the Delis-Kaplan Executive Functions Scale, Digit Span Backward task, M’s and N’s task, California Verbal Learning Test-SF, Boston Naming Test, semantic fluency (animals in one minute) and phonemic fluency (D-words in one minute).
Balas et al., 2010	Attention task; verbal fluency test; learning new verbal information	RAVLT; digit span; WAIS-III; Stroop word color test.
Uluduz et al., 2010	Ideomotor apraxia, ideational apraxia	MMSE, Mayo Clinic praxis test battery.
Brown et al., 2010	Global cognition, executive function	MMSE, DRS, FAB.

MMSE and the DSM-IV are probably not sufficiently sensitive to detect cognitive impairment in MSA.^15,41^ Based on this well-designed prospective study NNIPPS, DRS and FAB provided sensitive and comprehensive information on this issue. Moreover, Bak et al.^41^ showed that Addenbrooke's cognitive examination (ACE) could be as sensitive as DRS.

## Conclusion

Robust data shows that cognitive impairment in MSA is a frequent feature and should be actively pursued. MMSE and DSM-IV criteria for dementia, common tools used in initial clinical evaluation, are not sensitive enough to detect these abnormalities in MSA patients. In this context, comprehensive neuropsychological scales are useful, such as the DRS, FAB and ACE.

The physiopathology of cognitive impairment in this patient group is not well understood, but MSA-P most likely shares the same mechanism involved in others synucleinopathies. However, recent reports show that the cerebellum plays a role in global cognition and this could also be important in MSA-C.

Dementia is still considered a non-supporting feature for MSA diagnosis in the latest consensus. The evidence reviewed in this paper calls for a revision of MSA concepts and diagnostic criteria.
